# Efficacy and safety of PD-1/L1 inhibitors as first-line therapy for metastatic colorectal cancer: a meta-analysis

**DOI:** 10.3389/fimmu.2024.1425596

**Published:** 2024-07-19

**Authors:** Zhilong Huang, Chunyan Li, Yanping Huang, Weiming Liang, Haiyun Tao

**Affiliations:** The First Affiliated Hospital of Guangxi University of Science and Technology, Guangxi University of Science and Technology, Liuzhou, China

**Keywords:** PD-1 inhibitor, programmed cell death protein 1 inhibitor, colorectal cancer, objective response rate, bevacizumab, chemotherapy, mismatch repair-deficient, meta-analysis

## Abstract

**Objective:**

To evaluate the efficacy and safety of PD-1/L1 inhibitors as first-line therapy in metastatic colorectal cancer(mCRC).

**Method:**

Articles evaluating first-line PD-1/L1 inhibitors for mCRC were sought in four databases (Pubmed, Embase, Web of Science, and the Cochrane Library) from the inception of the databases until 11 November 2023. Meta-analyses were conducted to assess the rates of progression-free survival (PFS), overall survival (OS), complete response (CR), partial response (PR), stable disease (SD), progressive disease (PD), objective response rate (ORR), disease control rate (DCR), and grade ≥ 3 treatment-related adverse events (trAEs).

**Results:**

Totally nine studies were included for meta-analysis. A subgroup analysis was performed based on mismatch repair(MMR) status and regimens. In patients diagnosed with mismatch repair-deficient(dMMR) mCRC who received PD-1/L1 inhibitors as their first-line treatment, the ORR was 0.54 (95% CI, 0.39 to 0.68), the median PFS was 53.2 months, the Grade≥ 3 TRAEs rate was 0.33(95% CI, 0.12 to 0.60) and the median OS was not determined. For patients with proficient mismatch repair (pMMR) mCRC who underwent a combined treatment of PD-1/L1 inhibitors, anti-VEGF monoclonal antibody and chemotherapy as their first-line therapy, the ORR was 0.62 (95% CI, 0.56 to 0.68), the median PFS was 10.1 months, the median OS was 26.7 months, and the Grade≥ 3 TRAEs rate was 0.59(95% CI, 0.39 to 0.77).

**Conclusion:**

Our results revealed that the utilization of PD-1/L1 inhibitors as first-line therapy for dMMR mCRC yielded highly favorable outcomes, while maintaining an acceptable level of safety. Administering a combination of PD-1/L1 inhibitors, anti-VEGF monoclonal antibody, and chemotherapy as first-line treatment in patients with pMMR mCRC led to an improved ORR. However, there was no significant improvement in the long-term prognosis of the tumor.

**Systematic review registration:**

https://www.crd.york.ac.uk/prospero/display_record.php?ID=CRD42024506196, identifier CRD42024506196.

## 1 Introduction

Colorectal cancer (CRC) ranks as the third most prevalent malignant tumor globally, with over 2 million diagnosed cases in 2020. It is also the second leading cause of cancer-related mortality, resulting in nearly 1 million fatalities year (1). Based on the existing statistics, it is projected that the worldwide prevalence of colorectal cancer will rise by 60% by the year 2030, consequently leading to a significant escalation in the global economic burden (2). CRC that has dMMR or high microsatellite instability (MSI-H) is identified by a significant number of mutations in the tumor, which usually leads to an immunological response against the tumor in its surrounding environment (3–5). About 15% of all patients with colorectal cancer have deficient DNA mismatch repair, with 12% having sporadic instances and 3% having inherited cases. About 80% of sporadic dMMR colorectal cancer cases are attributed to methylation of the MLH1 gene promoter, while over 70% of hereditary cases are linked to germ-line mutations in the MLH1 and MSH2 genes (6–10). Antibodies against programmed cell death 1 or its ligand (PD-1/L1 inhibitors), such as pembrolizumab and nivolumab, has proven to be a viable treatment option for patients with metastatic colorectal cancer who have high microsatellite instability or deficient mismatch repair. This medication has shown success in cases when chemotherapy has not been beneficial (11, 12). In addition, preliminary data from the randomized, phase 3 KEYNOTE-177 trial comparing pembrolizumab to standard-of-care chemotherapy in patients with previously untreated mCRC with MSI-H/dMMR demonstrated that pembrolizumab provided improved health-related quality of life and superior progression-free survival as a first-line treatment (13, 14). The results provided evidence that led to the approval of pembrolizumab by the FDA and European Medicines Agency for the initial treatment of individuals with MSI-H/dMMR mCRC (15, 16).

Nevertheless, most metastatic colorectal tumors possess a proficient mismatch repair (pMMR) mechanism and exhibit microsatellite stability (MSS), rendering them inherently impervious to immune checkpoint inhibitors (17). In most cases of pMMR mCRC, there is a lack of immune response or an immune-excluded microenvironment. This is characterized by the absence or inactivity of CD8 T lymphocytes and decreased expression of checkpoint proteins on tumor cells (18–20). An attractive research technique is the combination of immune checkpoint inhibitors with antitumoral medicines that have immunomodulatory effects. The goal is to make these drugs more effective by boosting the immunogenicity of pMMR or MSS tumors (21). By stimulating CD8 T lymphocyte activation and immunogenic cell death, cytotoxic drugs are able to promote the release of neoantigens associated with tumors, leading to an immune-enriched microenvironment (22, 23). As demonstrated in a group of liver metastases removed following FOLFOXIRI-based regimens, this impact is anticipated to be more noticeable with upfront aggressive regimens, like a three-drug chemotherapy treatment (24). Bevacizumab improves the process of preparing and activating CD8 T lymphocytes by promoting the maturation of dendritic cells. It also increases the entry of CD8 T lymphocytes into tumors by normalizing the vasculature of the tumor. Additionally, it creates a tumor microenvironment that is conducive to immune responses (25). The AtezoTRIBE trial showed that incorporating atezolizumab into the initial treatment of FOLFOXIRI plus bevacizumab could potentially enhance the length of time without disease progression in individuals diagnosed with metastatic colorectal cancer (26).

A meta-analysis conducted by Yuegang Li et al (27) revealed that anti-PD-1/PD-L1 therapy in MSI-H/dMMR advanced CRC was linked to enhanced survival. However, it is important to note that the majority of studies did not focus on first-line therapy. In a meta-analysis conducted by He Jin et al (28), pembrolizumab was compared to other treatments for previously untreated, unresectable or metastatic microsatellite instability-high or mismatch repair-deficient colorectal cancer. The findings suggest that pembrolizumab is a highly effective and safe treatment for this population. However, the analysis did not mention other PD-1/L1 inhibitors. A protocol (29) was designed to provide a full pooled analysis of clinical trial data regarding immune checkpoint inhibitors for patients with microsatellite instability-high colorectal cancer, however, the findings have not been published. Therefore, we performed a meta-analysis to evaluate the effectiveness and safety of PD-1/L1 inhibitors when used as the first-line therapy for metastatic colorectal cancer.

## 2 Materials and methods

### 2.1 Search strategy

The current meta-analysis adhered to the 2020 guidelines set by the Preferred Reporting Project for Systematic Review and Meta-Analysis (PRISMA).The study has been registered at PROSPERO under the registration number CRD42024501740. A systematic search was conducted in four databases, namely PubMed, Embase, Web of Science, and the Cochrane Library, for literature published until November 11, 2023. The search strategy followed the PICOS principle and involved a combination of MeSH terms and free-text words. The specific search strategy used was: “PD-1/L1 inhibitor” AND “colorectal cancers” AND “trial”. **Supplementary Material 1** provided a comprehensive overview of the search record.

### 2.2 Inclusion and exclusion criteria

Inclusion criteria were as follows (1): patients diagnosed as untreated metastatic or advanced colorectal cancer (2); PD-1/L1 inhibitors as first-line therapy, with or without chemotherapy or other treatment (3); at least one of the following outcomes were reported: rates of complete response (CR), partial response (PR), stable disease (SD), progressive disease (PD), overall response rate (ORR), disease control rate (DCR), grade ≥ 3 treatment-related adverse events (trAEs) (4);Types of studies: randomized controlled studies, non-randomized controlled studies, single-arm trials, prospective studies or retrospective studies.

The exclusion criteria are as follows (1): other types of articles, such as case reports, publications, letters, comments, reviews, meta-analyses, editorials, animal studies, protocols, conference, etc (2); other cancers or diseases (3); not relevant (4); not first-line therapy (5); failed to extract data (6); duplicate patient cohort.

### 2.3 Selection of studies

The literature selection process, which involved removing duplicate entries, was conducted using EndNote (Version 20; Clarivate Analytics).The initial search was undertaken by two independent reviewers who deleted duplicate records, appraised the titles and abstracts for relevance, and categorized each study as either included or omitted. We arrived at a resolution through the attainment of consensus. Without an agreement among the parties involved, a third reviewer took on the role of a mediator.

### 2.4 Data extraction

The data was extracted by two reviewers independently. The extracted data included (1): Basic information of the study, including the first author, publication year, country, study design, sample size, and main outcomes (2); Baseline characteristics of study subjects, including number of patients, age, tumor type, and microsatellite status (3); The data analyzed included CR, PR, SD, PD, ORR, DCR, grade ≥ 3 trAEs rate, Kaplan-Meier curves for OS, and Kaplan-Meier curves for PFS. The discrepancy was resolved through the process of seeking advice from a third investigator.

### 2.5 Quality assessment

Two independent reviewers evaluated the quality assessment in the included trials. In this study, we employed the modified Jadad scale to assess the quality of randomized controlled trials. The single-arm trials were evaluated using methodological indicators from non-randomized studies(MINORS). In the event of any inconsistencies, the contentious findings were resolved by collective deliberation.

### 2.6 Statistical analysis

The analyses were conducted using Stata 12.0 and R version 4.3.1, which is copyrighted by The R Foundation for Statistical Computing in 2023. The analysis made use of the “meta” package and the IPDformKM package. The GetData Graph Digitizer software was utilized to extract data from articles that included Kaplan-Meier curves, and the individual data were subsequently reconstructed using the IPDformKM package. The approach developed by Guyot et al. was employed to reconstruct patient-specific data at an individual level (30). The comparison of continuous variables was conducted using the weighted mean difference (WMD) along with a 95% confidence interval (CI). The relative ratio (RR) with a 95% CI was employed to compare binary variables. The medians and interquartile ranges of continuous data were transformed into the mean and standard deviation. The statistical heterogeneity among the included studies was assessed using the Cochrane ‘Sq test and the I^2^ index. Given that the studies included in the analysis are derived from the public literature, it is often more reasonable to opt for the random effect model as the initial choice. A p value less than 0.05 was deemed to be statistically significant.

## 3 Results

### 3.1 Search results

**Figure 1:**
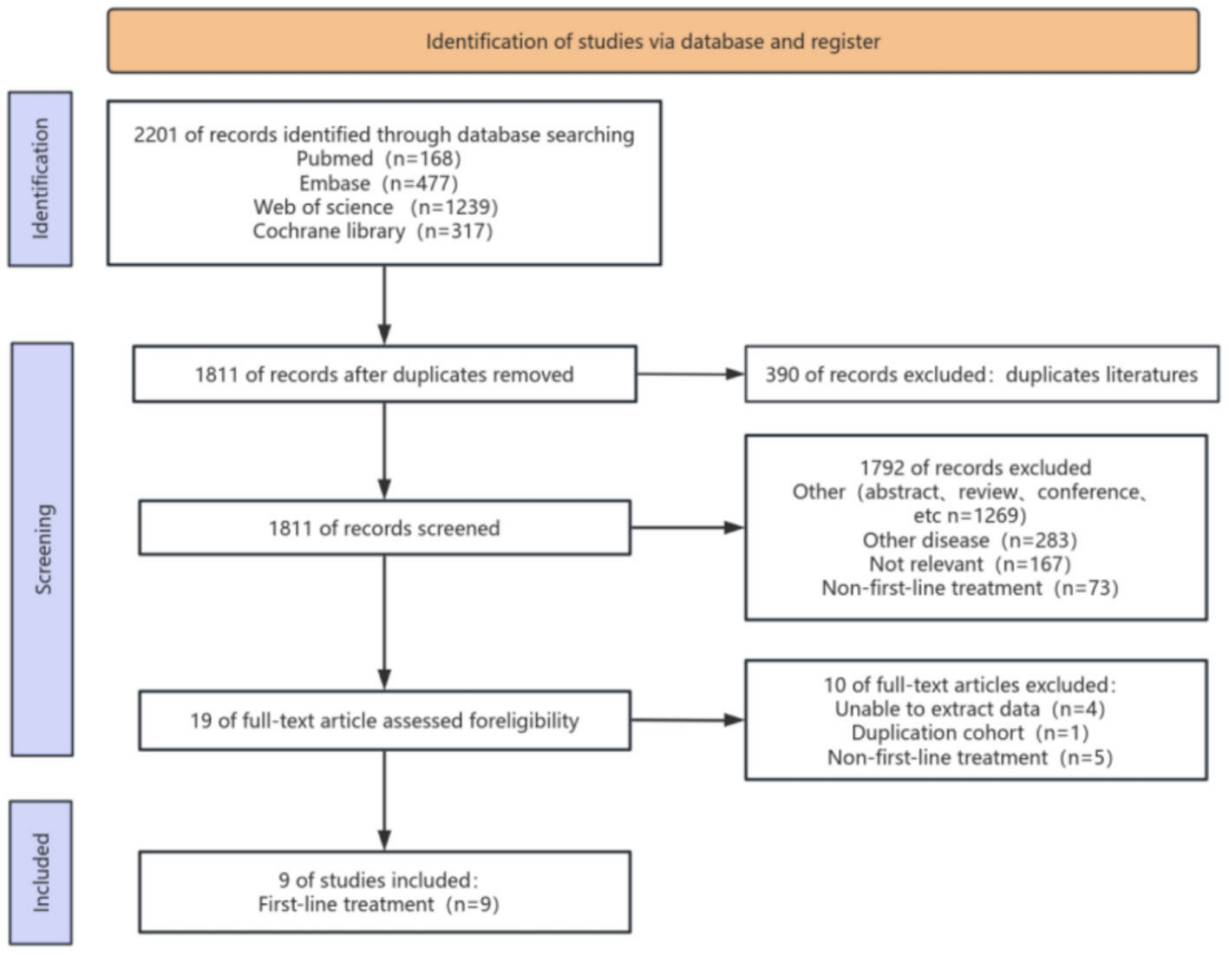
Flow chart of literature search strategies.

**Figure 1** illustrates the procedure of selecting and incorporating literature. We initially discovered a grand total of 2201 studies. After eliminating duplicate studies, only 1811 articles remained. After analyzing the titles and abstracts, a total of 1792 publications were deemed irrelevant and hence excluded. Upon thorough examination of the complete text, a total of 9 articles were ultimately selected for inclusion in this meta-analysis.

### 3.2 Patient characteristics and quality assessment

This meta-analysis comprised a total of nine articles, consisting of four randomized controlled trials, four single-arm studies. Total 821 patients with mCRC were included. The analysis was limited to the data of patients with mCRC who received first-line treatment with PD-1/L1 inhibitors. Within the trials analyzed, three of them (31–33) specifically focused on patients with dMMR mCRC who were treated with PD-1/L1 inhibitors (pembrolizumab or nivolumab in combination with low dose of ipilimumab), without the use of chemotherapy. These patients were categorized as subgroup A. Six further studies (26, 34–38) mostly investigated individuals with pMMR mCRC who were administered a combination of PD-1/L1 inhibitors, anti-VEGF antibody and chemotherapy. The patients were categorized as subgroup B. The results were combined using per protocol analysis and subgroup analysis because to variations in the literature included. For quality assessment, we employed the modified Jadad scale to evaluate the quality of RCT literature. The single-arm trials were evaluated using MINORS. All studies were of high quality. **Table 1** contains the specific information regarding patient characteristics and quality assessment.

**Table 1:** Patient characteristics and quality assessment of included studies and patients.

Author, year	Country	Design	Registration ID	Subgroup	PD-1/PD-L1 inhibitors	anti-VEGF antibody	Chemotherapy	Cases	dMMR/pMMR/ unknown	Median year	Male%	Quality
Bahar 2023 (31)	USA	single-arm	NA	A	Pembrolizumab	NA	NA	41	41/0/0	81.0	29.0	13
Luis 2022 (32)	USA	RCT	NCT02563002	A	Pembrolizumab	NA	NA	153	153/0/0	63.0	46.4	7
Heinz 2022 (33)	USA	single-arm	NCT02060188	A	Nivolumab, low dose ipilimumab	NA	NA	45	45/0/0	66.0	51.0	14
Carlotta 2022 (26)	Italy	RCT	NCT03721653	B	Atezolizumab	Bevacizumab	FOLFOXIRI	145	8/132/5	60.0	57.0	5
Xuefeng Fang 2023 (38)	China	single-arm	NCT04194359	B	Sintilimab	Bevacizumab	Oxaliplatin, Capecitabine	25	0/25/0	60.0	72.0	14
Jason M 2022 (35)	USA	RCT	NCT03050814	B	Avelumab	Bevacizumab	FOLFOX, CEA-targeted vaccine	16	0/16/0	NA	68.8	5
J Tab 2022 (34)	Spain	RCT	NCT02291289	B	Atezolizumab	Bevacizumab	FOLFOX	297	5/247/45	62.0	59.6	6
Marion 2023 (37)	France	single-arm	NCT03202758	B	Durvalumab	Tremelimumab	mFOLFOX6	57	3/54/0	63.6	42.1	13
Joseph 2022 (36)	Germany	single-arm	NCT03174405	B	Avelumab	Cetuximab	FOLFOX	43	3/36/3	62.0	66.7	13

RCT, randomized controlled trial; NA, not available; dMMR, mismatch repair deficiency; pMMR, proficient mismatch repair; FOLFOXIRI, Fluorouracil/calcium folinate, oxaliplatin and irinotecan; FOLFOX, Oxaliplatin, calcium folinate, fluorouracil.

### 3.3 Radiographic Response (CR, PR, SD, PD, ORR and DCR

**Figure 2:**
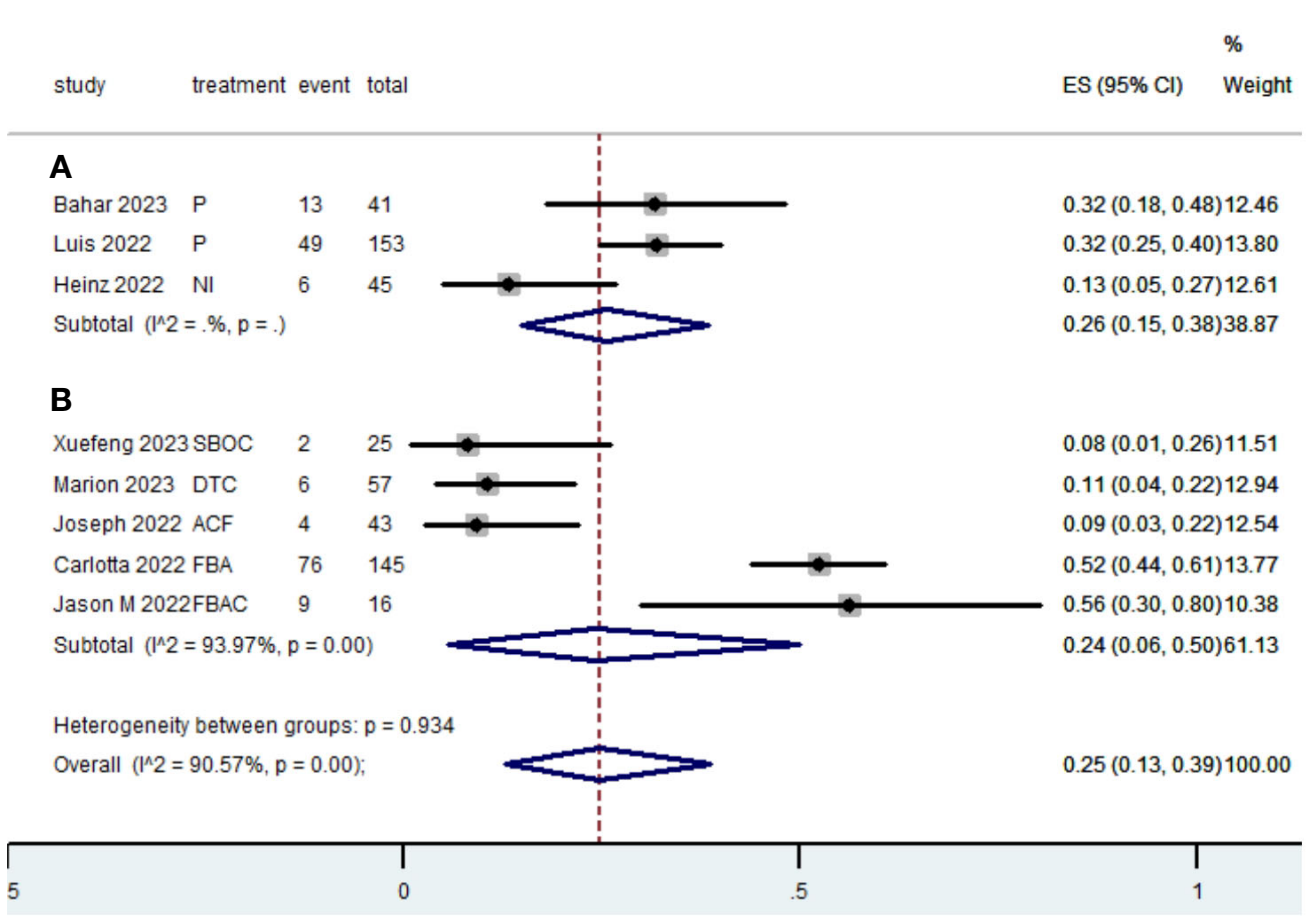
Forest plot of the meta-analysis for CR (Subgroup **A**: dMMR mCRC; Subgroup **B**: pMMR mCRC).

**Figure 3:**
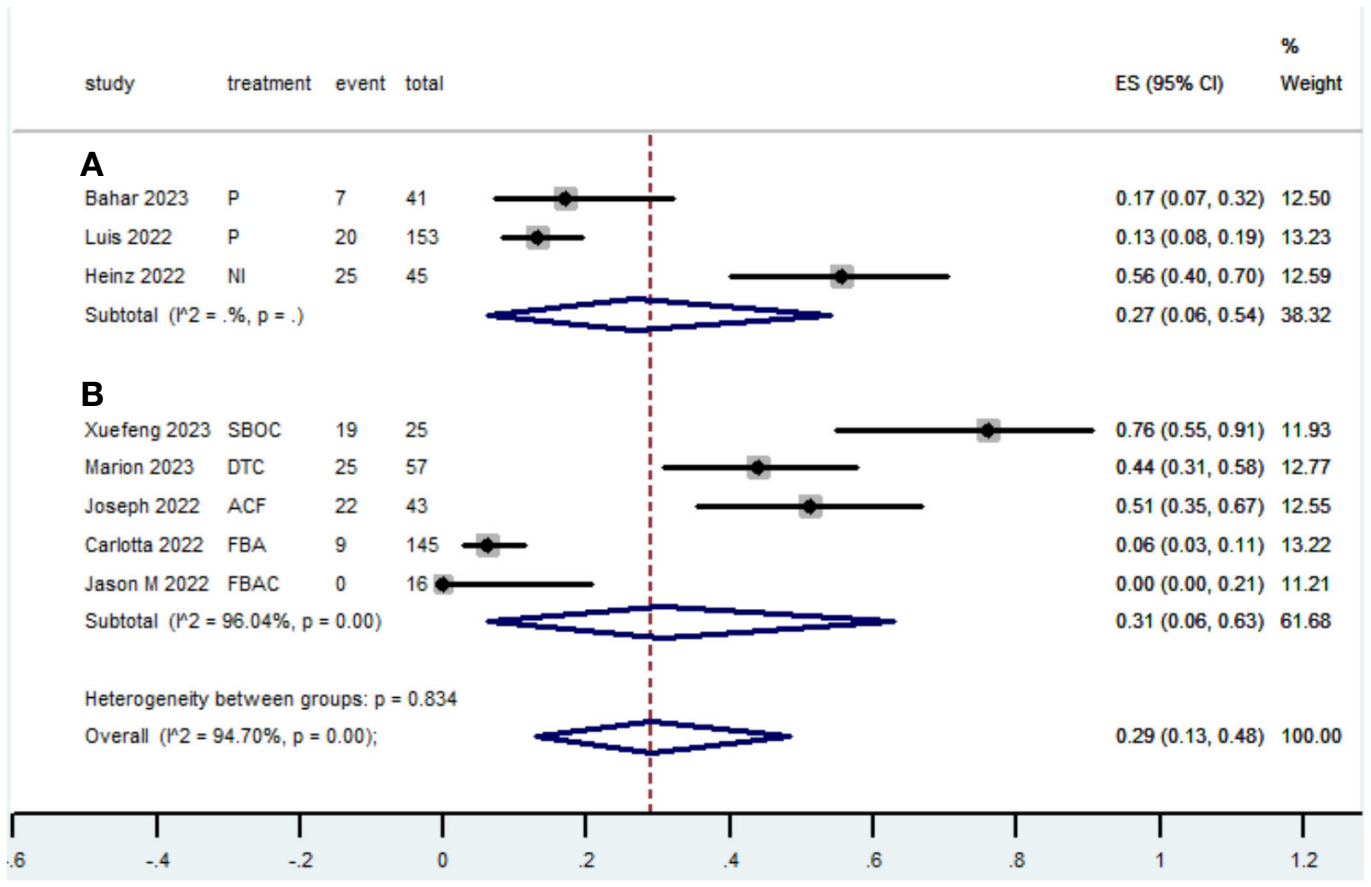
Forest plot of the meta-analysis for PR (Subgroup **A**: dMMR mCRC; Subgroup **B**: pMMR mCRC).

**Figure 4:**
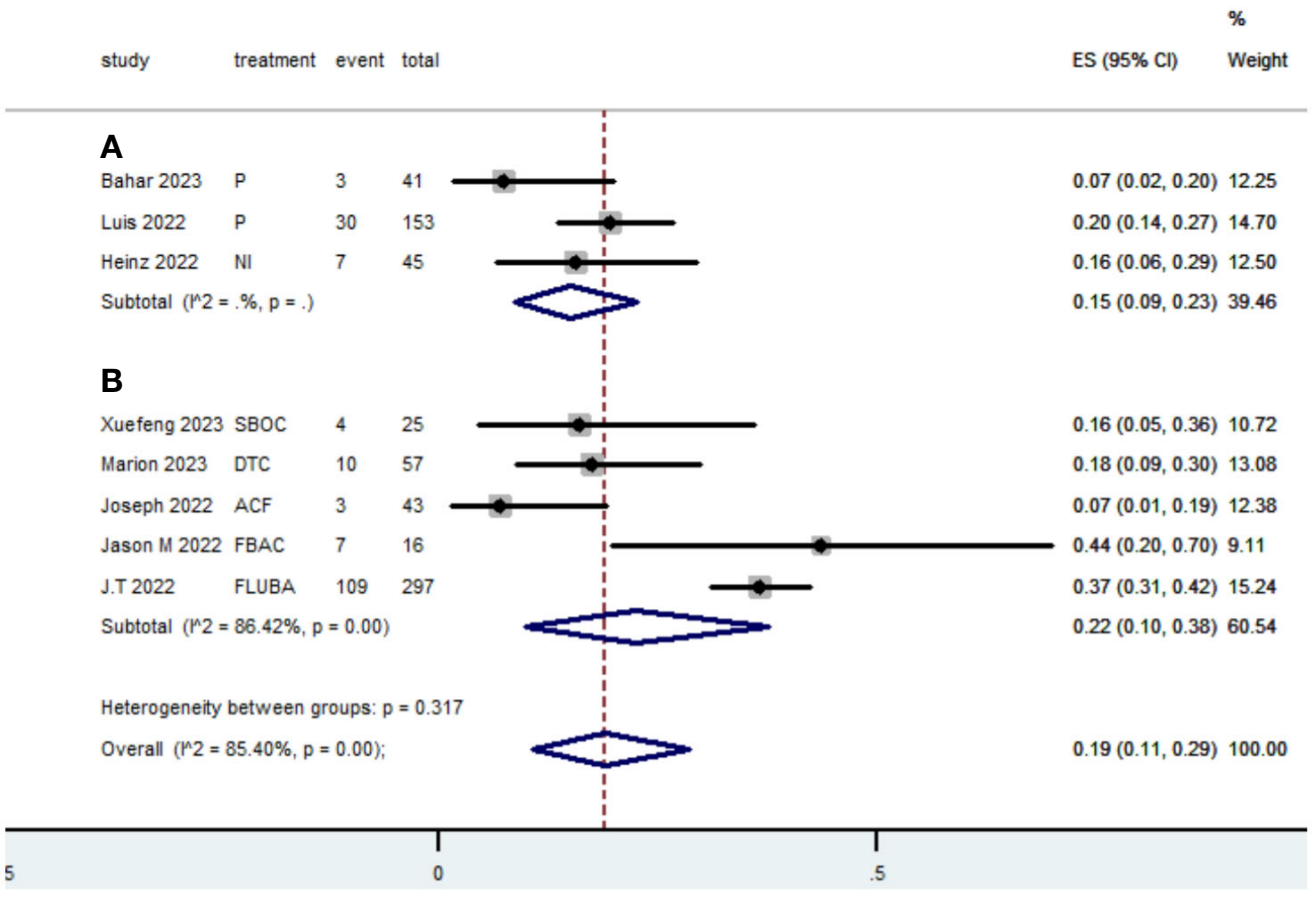
Forest plot of the meta-analysis for SD (Subgroup **A**: dMMR mCRC; Subgroup **B**: pMMR mCRC).

**Figure 5:**
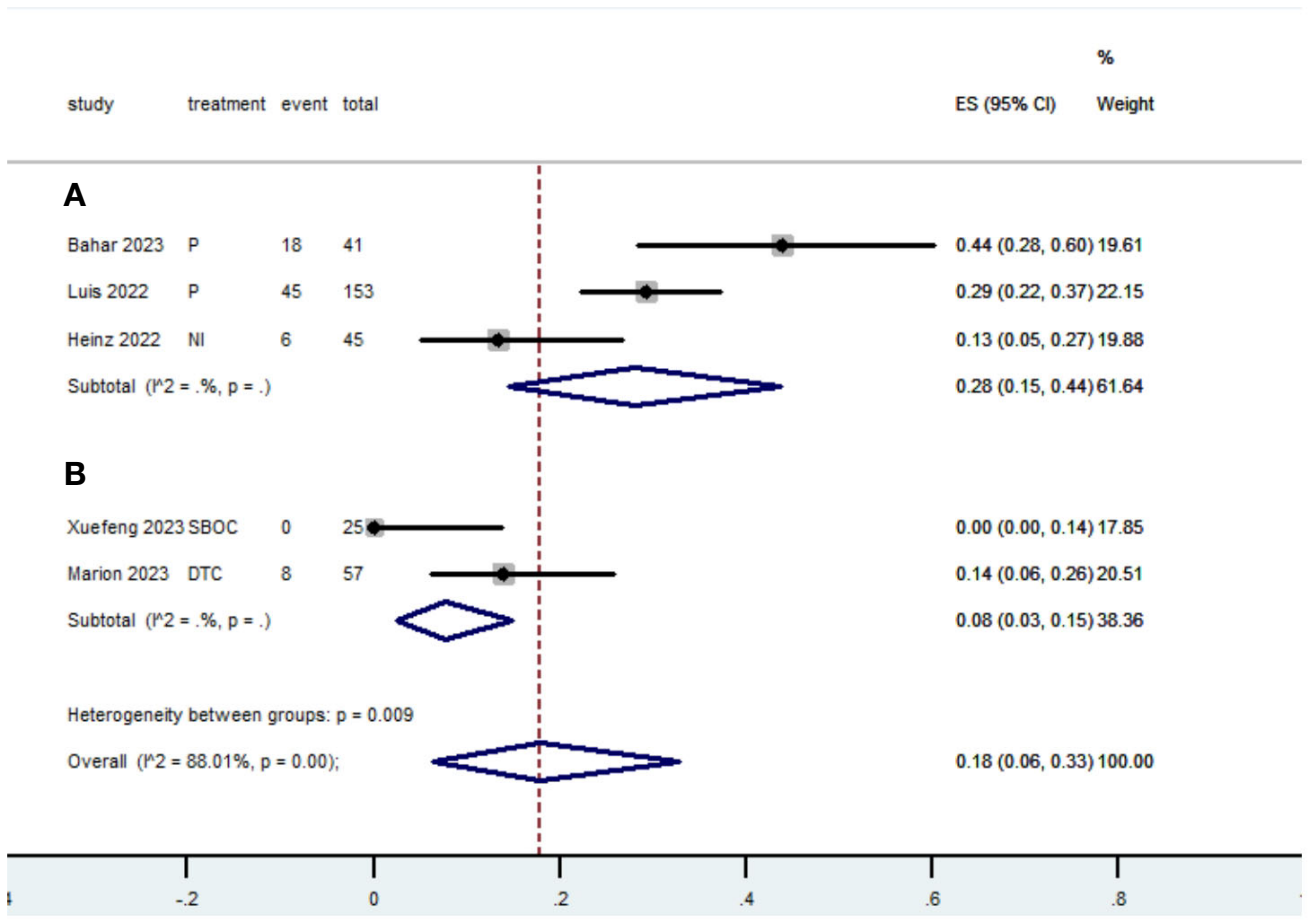
Forest plot of the meta-analysis for PD (Subgroup **A**: dMMR mCRC; Subgroup **B**: pMMR mCRC).

**Figure 6:**
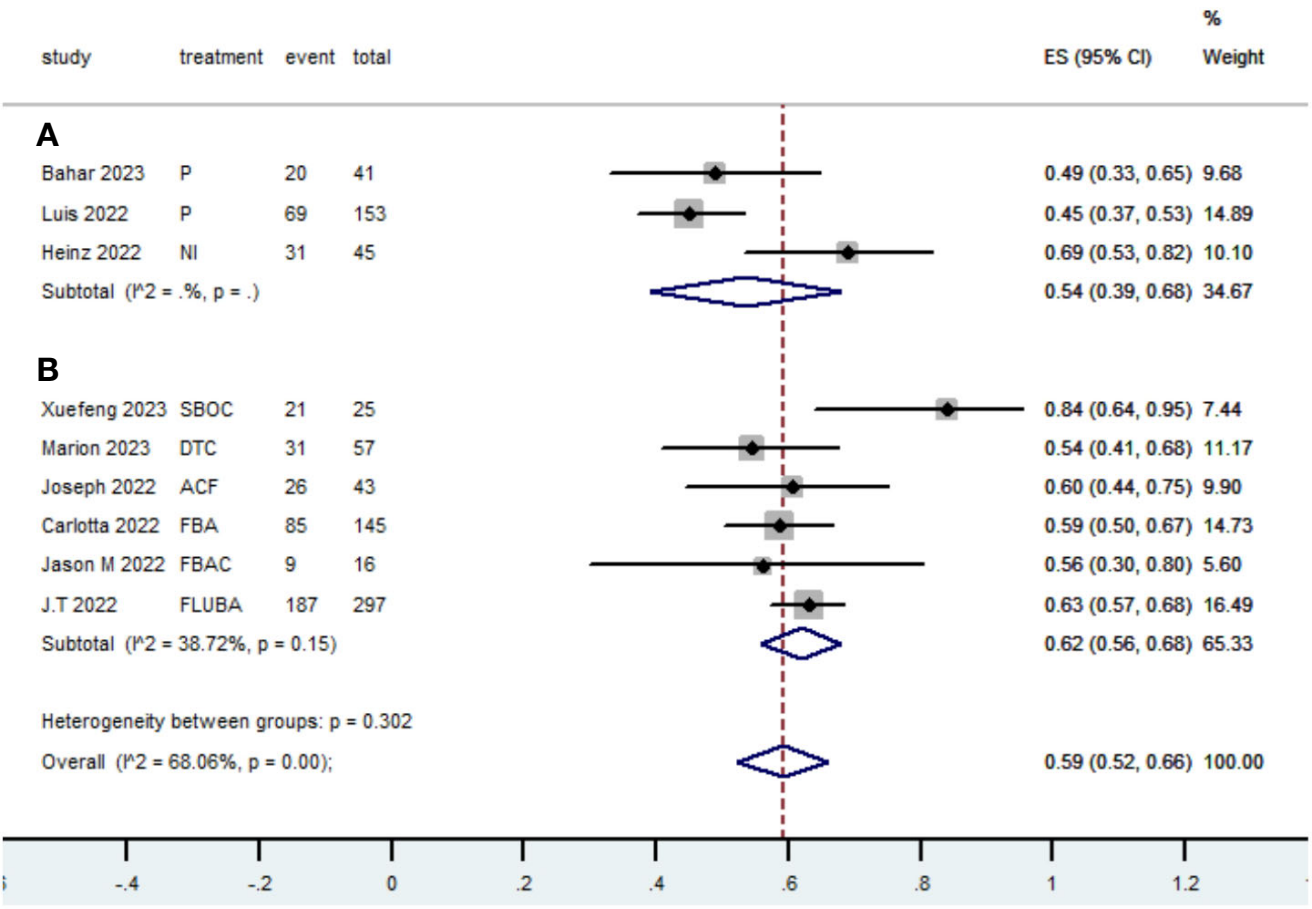
Forest plot of the meta-analysis for ORR (Subgroup **A**: dMMR mCRC; Subgroup **B**: pMMR mCRC).

**Figure 7:**
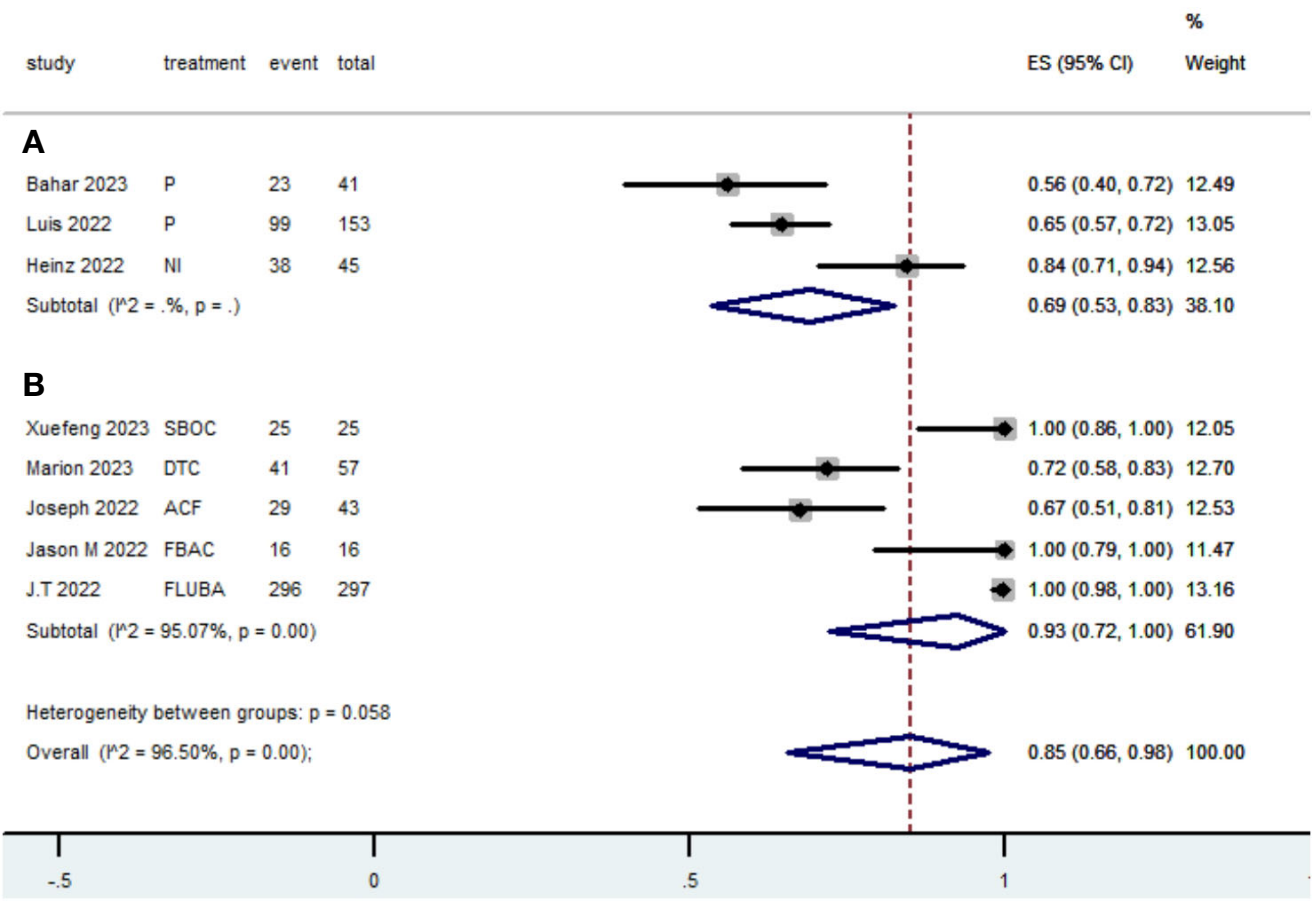
Forest plot of the meta-analysis for DCR (Subgroup **A**: dMMR mCRC; Subgroup **B**: pMMR mCRC).

**Table 2** presents a summary of the radiographic response outcomes for two groups. The radiographic response for patients with dMMR mCRC who were treated with PD-1/L1 inhibitors as their initial therapy were as follows:CR-0.26 (95% CI, 0.15 to 0.38)(**Figure 2**), PR-0.27 (95% CI, 0.06 to 0.54) (**Figure 3**), SD-0.15 (95% CI, 0.09 to 0.23) (**Figure 4**), PD-0.28 (95% CI, 0.15 to 0.44) (**Figure 5**), ORR-0.54 (95% CI, 0.39 to 0.68) (**Figure 6**) and DCR-0.69 (95% CI, 0.53 to 0.83) (**Figure 7**). Besides, the radiographic response for patients with pMMR mCRC who received a combination of PD-1/L1 inhibitors, anti-VEGF antibody and chemotherapy as their first-line therapy were as follows:CR-0.24 (95% CI, 0.06 to 0.50) (**Figure 2**), PR-0.31 (95% CI, 0.06 to 0.63) (**Figure 3**), SD-0.22 (95% CI, 0.10 to 0.38) (**Figure 4**), PD-0.08(95% CI, 0.03 to 0.15) (**Figure 5**), ORR-0.62 (95% CI, 0.56 to 0.68)(**Figure 6**) and DCR-0.93(95% CI, 0.72 to 0.10) (**Figure 7**).

**Table 2:** The results of the meta-analysis for pCR, pPR, SD, DCR, PD, ORR and Grade≥ 3 TRAEs rate.

Outcomes	Patients	No. Of studies	Overall effect size	95% CI of overall effect
CR				
Subgroup A	68	3	0.26	0.15-0.38
Subgroup B	97	5	0.24	0.06-0.50
Overall pooled CR	165	8	0.25	0.13-0.39
PR				
Subgroup A	52	3	0.27	0.06-0.54
Subgroup B	75	5	0.31	0.06-0.63
Overall pooled PR	127	8	0.29	0.13-0.48
SD				
Subgroup A	40	3	0.15	0.09-0.23
Subgroup B	133	5	0.22	0.10-0.38
Overall pooled SD	173	8	0.19	0.11-0.29
PD				
Subgroup A	69	3	0.28	0.15-0.44
Subgroup B	8	2	0.08	0.03-0.15
Overall pooled PD	77	5	0.18	0.06-0.33
ORR				
Subgroup A	120	3	0.54	0.39-0.68
Subgroup B	359	6	0.62	0.56-0.68
Overall pooled ORR	479	9	0.59	0.52-0.66
DCR				
Subgroup A	160	3	0.69	0.53-0.83
Subgroup B	407	5	0.93	0.72-1.00
Overall pooled DCR	567	8	0.85	0.66-0.98
Grade≥ 3 TRAEs rate				
Subgroup A	105	3	0.33	0.12-0.60
Subgroup B	266	5	0.59	0.39-0.77
Overall pooled Grade≥ 3 TRAEs rate	371	8	0.49	0.35-0.63

Subgroup A: patients diagnosed with dMMR mCRC who received PD-1/L1 inhibitors as their first-line treatment.

Subgroup B: patients with pMMR mCRC who underwent a combined treatment of PD-1/L1 inhibitors, anti-VEGF monoclonal antibody and chemotherapy as their first-line therapy.

CR, complete response; PR, partial response; SD, stable disease; PD, progressive disease; ORR, Objective Response Rate; DCR, Disease Control Rate; TRAEs, Treatment-Related Adverse Events.

### 3.4 PFS and OS

**Figure 8:**
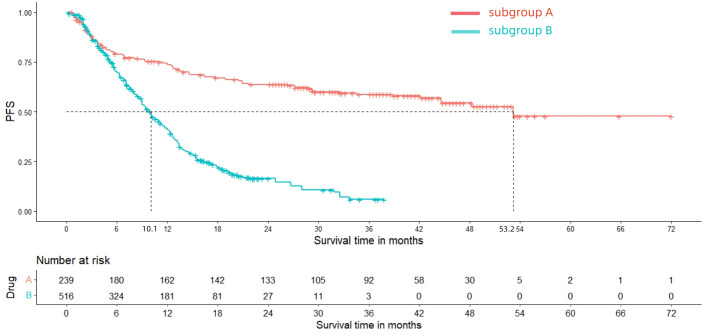
Kaplan-Meier curves for PFS (Subgroup **A**: dMMR mCRC; Subgroup **B**: pMMR mCRC).

**Figure 9:**
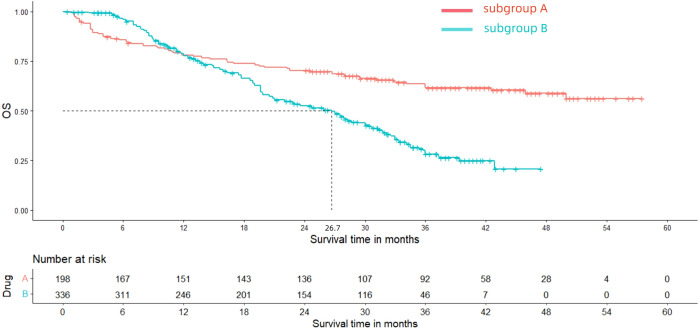
Kaplan-Meier curves for OS (Subgroup **A**: dMMR mCRC; Subgroup **B**: pMMR mCRC).

After reconstructing the cohort, we performed an additional assessment of PFS and OS by Kaplan-Meier curve. **Figures 8**, **9** displayed the PFS and OS outcomes of patients. For patients with dMMR mCRC who were treated with PD-1/L1 inhibitors as their initial therapy, the median PFS was 53.2 months. Significantly, the median OS were not determined due to the short follow-up period of the studies included. Besides, for patients with pMMR mCRC who received a combination of PD-1/L1 inhibitors, anti-VEGF antibody and chemotherapy as their first-line therapy, the median PFS and OS were 10.1 months and 26.7months, respectively. In addition, we presented regular updates on PFS and OS for each group at 6-month intervals, ranging from 0 to 36 months. These updates are shown in **Table 3**.

**Table 3:** The results of OS and PFS for each group at 6-month intervals.

Outcomes	6 months	12 months	18 months	24 months	30 months	36 months
OS						
Subgroup A	86.17%	79.08%	74.38%	72.17%	66.94%	63.21%
Subgroup B	96.56%	78.68%	66.95%	53.12%	43.57%	28.67%
PFS						
Subgroup A	79.68%	74.66%	67.42%	64.04%	60.28%	59.25%
Subgroup B	70.11%	41.67%	22.87%	16.98%	11.23%	6.54%

Subgroup A: patients diagnosed with dMMR mCRC who received PD-1/L1 inhibitors as their first-line treatment.

Subgroup B: patients with pMMR mCRC who underwent a combined treatment of PD-1/L1 inhibitors, anti-VEGF monoclonal antibody and chemotherapy as their first-line therapy.

OS, overall survival.

PFS, progression-free survival.

### 3.5 Grade≥ 3 TRAEs rate

**Figure 10:**
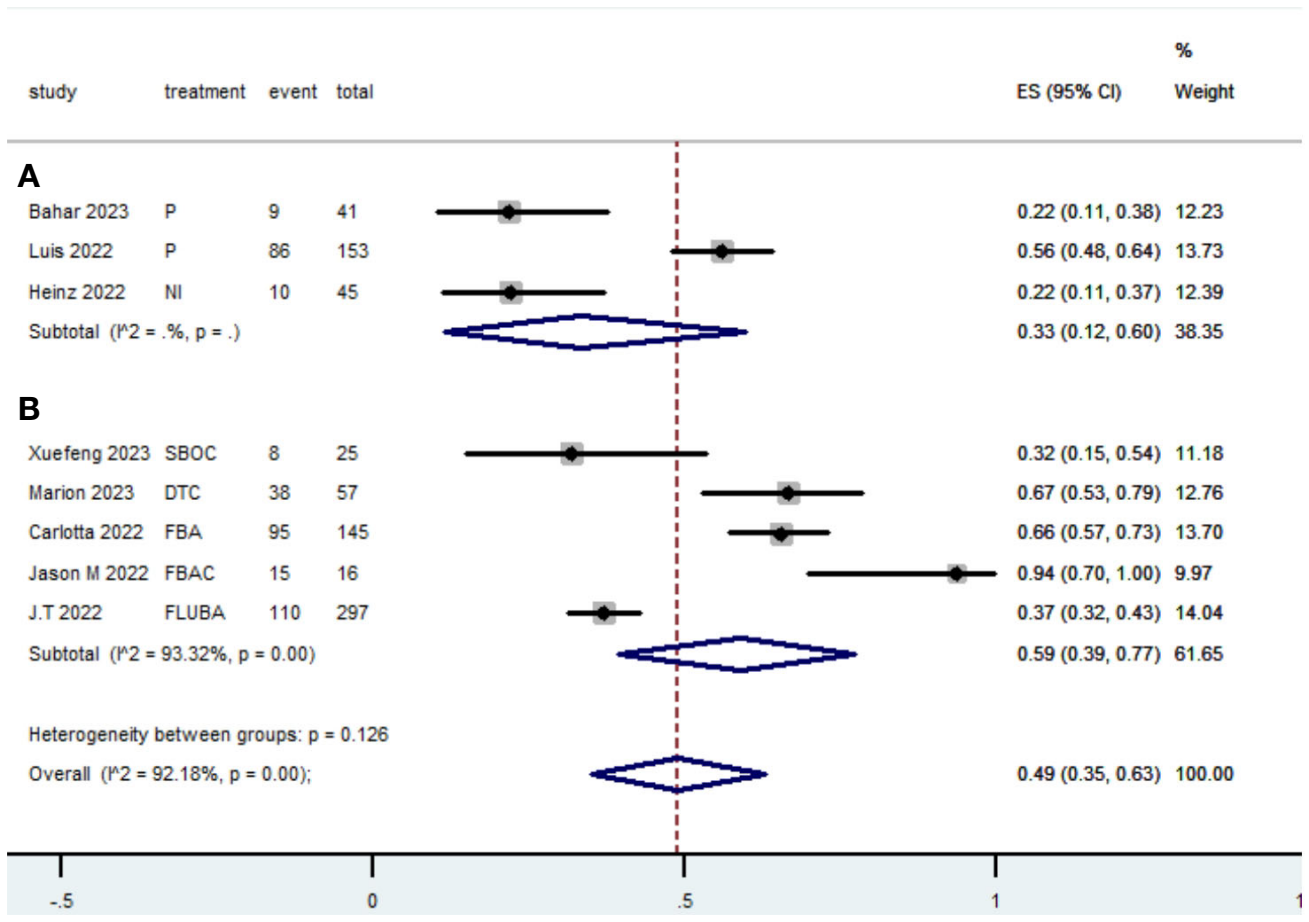
Forest plot of the meta-analysis for Grade≥ 3 TRAEs rate (Subgroup **A**: dMMR mCRC; Subgroup **B**: pMMR mCRC).

The Grade≥ 3 TRAEs rate was found to be 0.33(95% CI, 0.12 to 0.60) (**Table 2**; **Figure 10**) for patients with dMMR mCRC who were treated with PD-1/L1 inhibitors as their initial therapy. For patients with pMMR mCRC who received a combination of PD-1/L1 inhibitors, anti-VEGF antibody and chemotherapy as their first-line therapy, the Grade≥ 3 TRAEs rate was 0.59(95% CI, 0.39 to 0.77) (**Table 2**; **Figure 8**).

## 4 Discussion

To our knowledge, this study represents the first meta-analysis conducted to evaluate the effectiveness and safety of PD-1/L1 inhibitors as first-line treatment for mCRC. Previous studies indicated that individuals diagnosed with dMMR mCRC who used chemotherapy as their initial treatment had unsatisfactory outcomes, with a median overall survival ranging from 13.6 to 21.5 months (13, 39–42). While the FDA and European Medicines Agency have suggested PD-1/L1 inhibitors as the first-line therapy for individuals with dMMR mCRC (15, 16, 43, 44), there is currently a scarcity of data about the use of PD-1/L1 inhibitors as the first-line therapy for dMMR mCRC. Our study’s findings suggest that PD-1/L1 inhibitors offer a significant therapeutic benefit compared to chemotherapy when used as the first-line treatment. In addition, PD-1/L1 inhibitors demonstrated an acceptable level of safety. The production of tumor-specific antigens is crucial for the long-lasting effectiveness of the immune response against tumors, since it promotes the generation of T cells that specifically target the tumor. Recent research indicates that tumors containing a higher quantity of new antigens are more likely to provoke an immune response, making them more suitable for immunotherapy. Microsatellite-instable (MSI) colorectal tumors, characterized by mutations in the mismatch repair gene, produce 10 to 50 times greater amounts of neoantigens compared to microsatellite-stable (MSS) colorectal cancers that lack these abnormalities (4). The heightened presentation of antigens in individuals with MSI (as opposed to MSS) colon cancer is linked to a notable increase in the infiltration of T-cells and improved prognoses. Our findings indicate that patients with dMMR mCRC who received first-line PD-1/L1 immunosuppressants in standard clinical practice experienced a meaningful increase in survival. However, it is worth noting that a considerable proportion of patients (28%) had progressive disease as their best response, indicating that some patients with dMMR mCRC may exhibit an inherent resistance to anti-PD-1/L1 monotherapy.

The majority of tumors in patients with metastatic colorectal cancer (mCRC) are pMMR/MSS tumors (45). The recommended treatment for individuals with metastatic colorectal cancer (mCRC) involves chemotherapy protocols that include fluorouracil, oxaliplatin, and/or irinotecan, together with medications that target angiogenesis (bevacizumab) or the epidermal growth factor receptor (cetuximab) (46, 47). Previous literature (48, 49) has demonstrated that utilizing FOLFOX-based chemotherapy in combination with the anti-VEGF monoclonal antibody bevacizumab as the initial treatment for mCRC is linked to a median PFS of around 10 months and a median OS of 25-29 months. Additionally, this treatment approach has shown an ORR ranging from 44.7% to 55.2%. Compared with standard therapy, our findings indicated that the combined use of PD-1/L1 inhibitors, anti-VEGF antibody, and chemotherapy as the initial treatment showed significant potential in suppressing tumor growth. However, it did not result in significant improvements in PFS or OS.

The enhanced ORR suggests that pMMR cancer does not exhibit complete resistance to immunotherapy. The future research is concentrated toward identifying potential biomarkers or indicators that can differentiate between responders and non-responders to immunotherapy in CRC, specifically for pMMR/MSS tumors. Identifying these indicators could enable categorizing patients based on their characteristics, which would help decrease the negative effects, expenses, and potentially assign people who do not respond to other treatment methods. Several studies have documented the investigation of factors that can predict the effectiveness of PD-1/L1 inhibitors when added to initial treatment in pMMR mCRC. An intended subgroup analysis of progression-free survival (PFS) in MODUL cohort 2 (34) revealed no notable in various factors between the experimental and control groups. These factors include age, sex, region, tumor response at the end of initial treatment period, baseline ECOG status, AJCC/UICC stage at diagnosis, prior systematic adjuvant therapy, number of metastatic sites at baseline, presence of liver metastatic sites at baseline, cancer type, tumor colon location, and initial diagnosis. The AVETUX trial (36) found that, in a multilinear correlation analysis, the only parameters that showed a link with response to therapy were the diversity and clonality of tumor-infiltrating lymphocytes. Carlotta et al (26) undertook a *post-hoc* analysis to investigate the correlation between treatment and significant baseline characteristics in a sample of patients with pMMR tumors. A noteworthy correlation was found between TMB and immunoscore IC in relation to the treatment group. They recommended that forthcoming trials investigating the inclusion of immune checkpoint inhibitors in first-line therapy should exclusively involve patients with high TMB, high immunoscore IC tumors, or both. Xuefeng Fang et al (38) performed an exploratory analysis of biomarkers, which revealed that certain patients with RAS mutations and microsatellite stable (MSS) status transitioned into a “immune-hot” subtype during therapy. They proposed that the specific processes behind these observations warrant additional investigation. Marion Thibaudin et al. (37) found that individuals who responded well to treatment had a larger tumor mutational burden and reduced genomic instability. Additionally, their integrated transcriptome study revealed that a strong immunological signature and low epithelial-mesenchymal transition were linked to improved outcomes. Immunomonitoring revealed the activation of certain T cells in the blood that target neoantigens, NY-ESO1, and TERT. This immune response was found to be related with improved PFS.

Currently, scientists are doing thorough investigations on the predictive significance of POLE mutations for immunotherapy. The POLE gene is situated on chromosome 12q24.33 and encodes a crucial subunit of DNA polymerase, which plays a vital role in DNA replication and repair( (50)). POLE mutations are associated with additional positive prognostic variables, including elevated PD-L1 expression, high TMB, and infiltration of CD8+ cells in the TME (51, 52). According to a report (53), tumors with a POLE mutation (POLEmt) consistently had a higher density of CD8+ T cells compared to tumors without the mutation (POLEwt). This was observed in both endometrial cancer (59.4 vs. 24.7 CD8+ cells per HPF, p = 0.11) and colorectal intraepithelial neoplasia (59.4 vs. 14.8 CD8+ cells per HPF, p = 0.029), as well as colorectal cancer (154.9 vs. 34.0 CD8+ cells per HPF, p value not provided). Domingo et al. also discovered a significant presence of CD8+ T-cells in POLEmt colorectal cancer (54). In a study of 37 patients with POLEmt endometrial cancer, 29.6% of them showed PD-L1 expression greater than 1%, whereas 27.8% had intratumoral T-cell infiltrates (55). Howitt et al. reported that PD-L1 expression was observed in 84% of POLEmt endometrial cancer cases with a level more than 10%. Additionally, the average number of CD8+TIL per high-power field (HPF) was 32.8 (56). A study conducted by Yang Fu et al. provided substantial evidence in favor of the efficacy of immune-combined therapy for advanced non-small cell lung cancer (NSCLC) patients with POLE mutation, including those with brain metastases (57).

Our study has clear advantages. First, this study is the inaugural meta-analysis to evaluate the effectiveness and safety of PD-1/L1 inhibitors as the initial therapy for mCRC. Second, the IPDformKM package was utilized to reconstruct Kaplan-Meier curves for OS and PFS, providing a clear and comprehensible representation of the oncological results. It offers evidence to guide clinical practice in the first-line treatment of patients with mCRC. Without a doubt, our study has certain limitations. At first, the sample size was rather diminutive. The analysis encompassed a mere nine trials including 821 persons diagnosed with mCRC who received PD-1/L1 inhibitors as their primary treatment. Secondly, the incorporation of single-arm clinical studies led to indirect comparisons across various treatment regimens. Furthermore, there was notable diversity across the nine studies in terms of research methodology, patient characteristics, and treatment regimens. Besides, some studies have not yet reached their endpoint, resulting in a lack of long-term survival outcome data. Hence, the explain of our findings necessitates a certain degree of prudence.

In conclusion, the results of our study demonstrated that using PD-1/L1 inhibitors as the initial treatment for dMMR mCRC was extremely effective, with acceptable safety. In patients with pMMR mCRC, the administration of a combination of PD-1/L1 inhibitors, anti-VEGF monoclonal antibody, and chemotherapy as the initial treatment resulted in an enhanced ORR. However, there was no significant improvement in PFS or OS. Future research efforts should be focused on developing biomarkers or indicators that can effectively distinguish between individuals who respond positively to immunotherapy and those who do not in the context of mCRC, particularly for pMMR/MSS status. Given the limitations of our study, it is crucial to conduct further multicenter, randomized controlled trials and extend the duration of follow-up in order to give further validation for our result.

## Data availability statement

The original contributions presented in the study are included in the article/**Supplementary Material**. Further inquiries can be directed to the corresponding author.

## Author contributions

ZH: Conceptualization, Data curation, Formal analysis, Investigation, Writing – original draft. CL: Conceptualization, Data curation, Formal analysis, Investigation, Writing – original draft. YH: Software, Supervision, Validation, Visualization, Writing – original draft. WL: Software, Supervision, Validation, Visualization, Writing – review & editing. HT: Funding acquisition, Resources, Visualization, Writing – review & editing.
