# Supplementary material for: Efficacy and safety of PD-1/L1 inhibitors as first-line therapy for metastatic colorectal cancer: a meta-analysis
Source: Front Immunol. 2024 Jul 19;15:1425596. doi: 10.3389/fimmu.2024.1425596 (PMC11294095; doi:10.3389/fimmu.2024.1425596)
Supplement: Supplementary file 1 [file Table_1.docx]

| PubMed | | |
| --- | --- | --- |
| No. | Query | Results |
| #1 | (((((((((((((((PD-1 inhibitor[Title/Abstract]) OR (Pembrolizumab[Title/Abstract])) OR (Nivolumab[Title/Abstract])) OR (Toripalimab[Title/Abstract])) OR (Tislelizumab[Title/Abstract])) OR (Camrelizumab[Title/Abstract])) OR (GLS-010[Title/Abstract])) OR (Cemiplimab[Title/Abstract])) OR (Sintilimab[Title/Abstract])) OR (Zimberelimab[Title/Abstract])) OR (Prolgolimab[Title/Abstract])) OR (Dostarlimab[Title/Abstract])) OR (PD-L1 inhibitor[Title/Abstract])) OR (Atezolizumab[Title/Abstract])) OR (Durvalumab[Title/Abstract])) OR (Avelumab[Title/Abstract]) | 20500 |
| #2 | (((((((((((((((((Rectal Neoplasms[Title/Abstract]) OR (Neoplasm, Rectal[Title/Abstract])) OR (Rectal Neoplasm[Title/Abstract])) OR (Rectum Neoplasms[Title/Abstract])) OR (Neoplasm, Rectum[Title/Abstract])) OR (Rectum Neoplasm[Title/Abstract])) OR (Rectal Tumors[Title/Abstract])) OR (Rectal Tumor[Title/Abstract])) OR (Tumor, Rectal[Title/Abstract])) OR (Neoplasms, Rectal[Title/Abstract])) OR (Cancer of Rectum[Title/Abstract])) OR (Rectum Cancers[Title/Abstract])) OR (Rectal Cancer[Title/Abstract])) OR (Cancer, Rectal[Title/Abstract])) OR (Rectal Cancers[Title/Abstract])) OR (Rectum Cancer[Title/Abstract])) OR (Cancer, Rectum[Title/Abstract])) OR (Cancer of the Rectum[Title/Abstract]) | 35877 |
| #3 | (((((((((((((((Colorectal Neoplasms[Title/Abstract]) OR (Colorectal Neoplasm[Title/Abstract])) OR (Neoplasm, Colorectal[Title/Abstract])) OR (Neoplasms, Colorectal[Title/Abstract])) OR (Colorectal Tumors[Title/Abstract])) OR (Colorectal Tumor[Title/Abstract])) OR (Tumor, Colorectal[Title/Abstract])) OR (Tumors, Colorectal[Title/Abstract])) OR (Colorectal Cancer[Title/Abstract])) OR (Cancer, Colorectal[Title/Abstract])) OR (Cancers, Colorectal[Title/Abstract])) OR (Colorectal Cancers[Title/Abstract])) OR (Colorectal Carcinoma[Title/Abstract])) OR (Carcinoma, Colorectal[Title/Abstract])) OR (Carcinomas, Colorectal[Title/Abstract])) OR (Colorectal Carcinomas[Title/Abstract]) | 153492 |
| #4 | ((((((((((((((((((((Colonic Neoplasm[Title/Abstract]) OR (Neoplasm, Colonic[Title/Abstract])) OR (Neoplasms, Colonic[Title/Abstract])) OR (Colon Neoplasms[Title/Abstract])) OR (Colon Neoplasm[Title/Abstract])) OR (Neoplasm, Colon[Title/Abstract])) OR (Neoplasms, Colon[Title/Abstract])) OR (Cancer of Colon[Title/Abstract])) OR (Colon Cancers[Title/Abstract])) OR (Colon Cancer[Title/Abstract])) OR (Cancer, Colon[Title/Abstract])) OR (Cancers, Colon[Title/Abstract])) OR (Cancer of the Colon[Title/Abstract])) OR (Colonic Cancer[Title/Abstract])) OR (Cancer, Colonic[Title/Abstract])) OR (Cancers, Colonic[Title/Abstract])) OR (Colonic Cancers[Title/Abstract])) OR (Colon Adenocarcinoma[Title/Abstract])) OR (Adenocarcinoma, Colon[Title/Abstract])) OR (Adenocarcinomas, Colon[Title/Abstract])) OR (Colon Adenocarcinomas[Title/Abstract]) | 71435 |
| #5 | ((((((Chemotherapy[Title/Abstract]) OR (Therapy, Drug[Title/Abstract])) OR (Drug Therapies[Title/Abstract])) OR (Therapies, Drug[Title/Abstract])) OR (Chemotherapies[Title/Abstract])) OR (Pharmacotherapy[Title/Abstract])) OR (Pharmacotherapies[Title/Abstract]) | 497659 |
| #6 | ((((("Prospective Studies"[Mesh]) OR (Prospective Study[Title/Abstract])) OR (Studies, Prospective[Title/Abstract])) OR (Study, Prospective[Title/Abstract])) OR (‘’Prospective Comparative Study’’’[Title/Abstract])) OR ( Prospective[Title/Abstract]) | 978278 |
| #7 | ((("Retrospective Studies"[Mesh]) OR (Studies, Retrospective[Title/Abstract])) OR (Study, Retrospective[Title/Abstract])) OR (Retrospective Study[Title/Abstract]) | 1211042 |
| #8 | (randomized controlled trial [pt] OR controlled clinical trial [pt] OR randomized [tiab] OR placebo [tiab] OR clinical trials as topic [mesh:noexp] OR randomly [tiab] OR trial [ti]) NOT (animals [mh] NOT humans [mh]) | 1449157 |
| #9 | #2 OR #3 OR #4 | 232254 |
| #10 | #1 AND #9 | 639 |
| #11 | #6 OR #7 OR #8 | 3340640 |
| #12 | #10 AND #11 | 168 |

| Web of science | | |
| --- | --- | --- |
| No. | Query | Results |
| #1 | TS=(PD-1 inhibitor OR Pembrolizumab OR Nivolumab OR Toripalimab OR Tislelizumab OR Camrelizumab OR GLS-010 OR Cemiplimab OR Sintilimab OR Zimberelimab OR Prolgolimab OR Dostarlimab OR PD-L1 inhibitor OR Atezolizumab OR Durvalumab OR Avelumab) | 66391 |
| #2 | TS=(Rectal Neoplasms OR Neoplasm, Rectal OR Rectal Neoplasm OR Rectum Neoplasms OR Neoplasm, Rectum OR Rectum Neoplasm OR Rectal Tumors OR Rectal Tumor OR Tumor, Rectal OR Neoplasms, Rectal OR Cancer of Rectum OR Rectum Cancers OR Rectal Cancer OR Cancer, Rectal OR Rectal Cancers OR Rectum Cancer OR Cancer, Rectum OR Cancer of the Rectum) | 157117 |
| #3 | TS=(Colorectal Neoplasms OR Colorectal Neoplasm OR Neoplasm, Colorectal OR Neoplasms, Colorectal OR Colorectal Tumors OR Colorectal Tumor OR Tumor, Colorectal OR Tumors, Colorectal OR Colorectal Cancer OR Cancer, Colorectal OR Cancers, Colorectal OR Colorectal Cancers OR Colorectal Carcinoma OR Carcinoma, Colorectal OR Carcinomas, Colorectal OR Colorectal Carcinomas) | 378142 |
| #4 | TS=(Colonic Neoplasm OR Neoplasm, Colonic OR Neoplasms, Colonic OR Colon Neoplasms OR Colon Neoplasm OR Neoplasm, Colon OR Neoplasms, Colon OR Cancer of Colon OR Colon Cancers OR Colon Cancer OR Cancer, Colon OR Cancers, Colon OR Cancer of the Colon OR Colonic Cancer OR Cancer, Colonic OR Cancers, Colonic OR Colonic Cancers OR Colon Adenocarcinoma OR Adenocarcinoma, Colon OR Adenocarcinomas, Colon OR Colon Adenocarcinomas) | 365108 |
| #5 | (((((((TS=(Chemotherapy)) OR TS=(Therapy, Drug)) OR TS=(Drug Therapies)) OR TS=(Therapies, Drug)) OR TS=(Chemotherapy)) OR TS=(Chemotherapies)) OR TS=(Pharmacotherapy)) OR TS=(Pharmacotherapies) | 59825 |
| #6 | TS=(Prospective Studies OR Prospective Study OR Studies, Prospective OR Study, Prospective OR Prospective Comparative Study OR Prospective) | 1216666 |
| #7 | TS=(Retrospective Studies OR Studies, Retrospective OR Study, Retrospective OR Retrospective Study ) | 1494677 |
| #8 | TS=(randomized controlled trial OR controlled clinical trial OR randomized OR placebo OR clinical trials as topic OR randomly OR trial) | 3775414 |
| #9 | #2 OR #3 OR #4 | 680465 |
| #10 | #1 AND #9 | 4724 |
| #11 | #6 OR #7 OR #8 | 5937157 |
| #12 | #10 AND #11 | 1239 |

| Embase | | |
| --- | --- | --- |
| No. | Query | Results |
| #1 | 'pd-1 inhibitor' OR (('pd 1'/exp OR 'pd 1') AND ('inhibitor'/exp OR inhibitor)) OR pembrolizumab:ti,ab,kw OR nivolumab:ti,ab,kw OR toripalimab:ti,ab,kw OR tislelizumab:ti,ab,kw OR camrelizumab:ti,ab,kw OR 'gls 010':ti,ab,kw OR cemiplimab:ti,ab,kw OR sintilimab:ti,ab,kw OR zimberelimab:ti,ab,kw OR prolgolimab:ti,ab,kw OR dostarlimab:ti,ab,kw OR 'pd-l1 inhibitor':ti,ab,kw OR atezolizumab:ti,ab,kw OR durvalumab:ti,ab,kw OR avelumab:ti,ab,kw | 55988 |
| #2 | ‘Rectal Neoplasms’/exp OR ‘Neoplasm, Rectal’:ab,ti,kw OR ‘Rectal Neoplasm’:ab,ti,kw OR ‘Rectum Neoplasms’:ab,ti,kw OR ‘Neoplasm, Rectum’:ab,ti,kw OR ‘Rectum Neoplasm’:ab,ti,kw OR ‘Rectal Tumors’:ab,ti,kw OR ‘Rectal Tumor’:ab,ti,kw OR ‘Tumor, Rectal’:ab,ti,kw OR ‘Neoplasms, Rectal’:ab,ti,kw OR ‘Cancer of Rectum’:ab,ti,kw OR ‘Rectum Cancers’:ab,ti,kw OR ‘Rectal Cancer’:ab,ti,kw OR ‘Cancer, Rectal’:ab,ti,kw OR ‘Rectal Cancers’:ab,ti,kw OR ‘Rectum Cancer’:ab,ti,kw OR ‘Cancer, Rectum’:ab,ti,kw OR ‘Cancer of the Rectum’:ab,ti,kw | 92243 |
| #3 | ‘Colorectal Neoplasms’/exp OR ‘Colorectal Neoplasm’:ab,ti,kw OR ‘Neoplasm, Colorectal’:ab,ti,kw OR ‘Neoplasms, Colorectal’:ab,ti,kw OR ‘Colorectal Tumors’:ab,ti,kw OR ‘Colorectal Tumor’:ab,ti,kw OR ‘Tumor, Colorectal’:ab,ti,kw OR ‘Tumors, Colorectal’:ab,ti,kw OR ‘Colorectal Cancer’:ab,ti,kw OR ‘Cancer, Colorectal’:ab,ti,kw OR ‘Cancers, Colorectal’:ab,ti,kw OR ‘Colorectal Cancers’:ab,ti,kw OR ‘Colorectal Carcinoma’:ab,ti,kw OR ‘Carcinoma, Colorectal’:ab,ti,kw OR ‘Carcinomas, Colorectal’:ab,ti,kw OR ‘Colorectal Carcinomas’:ab,ti,kw | 497316 |
| #4 | ‘Colonic Neoplasm’/exp OR ‘Neoplasm, Colonic’:ab,ti,kw OR ‘Neoplasms, Colonic’:ab,ti,kw OR ‘Colon Neoplasms’:ab,ti,kw OR ‘Colon Neoplasm’:ab,ti,kw OR ‘Neoplasm, Colon’:ab,ti,kw OR ‘Neoplasms, Colon’:ab,ti,kw OR ‘Cancer of Colon’:ab,ti,kw OR ‘Colon Cancers’:ab,ti,kw OR ‘Colon Cancer’:ab,ti,kw OR ‘Cancer, Colon’:ab,ti,kw OR ‘Cancers, Colon’:ab,ti,kw OR ‘Cancer of the Colon’:ab,ti,kw OR ‘Colonic Cancer’:ab,ti,kw OR ‘Cancer, Colonic’:ab,ti,kw OR ‘Cancers, Colonic’:ab,ti,kw OR ‘Colonic Cancers’:ab,ti,kw OR ‘Colon Adenocarcinoma’:ab,ti,kw OR ‘Adenocarcinoma, Colon’:ab,ti,kw OR ‘Adenocarcinomas, Colon’:ab,ti,kw OR ‘Colon Adenocarcinomas’:ab,ti,kw | 209840 |
| #5 | 'chemotherapy'/exp OR chemotherapy OR 'therapy, drug':ti,ab,kw OR 'drug therapies':ti,ab,kw OR 'therapies, drug':ti,ab,kw OR chemotherapy:ti,ab,kw OR chemotherapies:ti,ab,kw OR pharmacotherapy:ti,ab,kw OR pharmacotherapies:ti,ab,kw | 1356336 |
| #6 | ‘Prospective Studies’:ab,ti,kw OR ‘Prospective Study’:ab,ti,kw OR ‘Studies, Prospective’:ab,ti,kw OR ‘Study, Prospective’:ab,ti,kw OR ‘Prospective Comparative Study’:ab,ti,kw OR ‘Prospective’:ab,ti,kw | 1118269 |
| #7 | ‘Retrospective Studies’:ab,ti,kw OR 'Studies, Retrospective’:ab,ti,kw OR ’Study, Retrospective’:ab,ti,kw OR ‘Retrospective Study’:ab,ti,kw | 366298 |
| #8 | 'randomized controlled trial'/exp OR 'randomized controlled trial':ti,ab,it OR 'randomized':ti,ab,it OR 'randomised':ti,ab,it OR 'randomization':ti,ab,it OR 'randomisa- tion':ti,ab,it OR rct:ti,ab,it OR 'randomly':ti,ab,it OR pla-cebo:ti,ab,it | 1747993 |
| #9 | #2 OR #3 OR #4 | 512239 |
| #10 | #1 AND #9 | 3322 |
| #11 | #6 OR #7 OR #8 | 2986617 |
| #12 | #10 AND #11 | 477 |

| cochrane library | | |
| --- | --- | --- |
| No. | Query | Results |
| #1 | (PD-1 inhibitor OR Pembrolizumab OR Nivolumab OR Toripalimab OR Camrelizumab OR GLS-010 OR Cemiplimab OR Sintilimab OR Zimberelimab OR Prolgolimab OR Dostarlimab OR PD-L1 inhibitor OR Atezolizumab OR Durvalumab OR Avelumab):ab,ti,kw | 8870 |
| #2 | (Rectal Neoplasms OR Neoplasm, Rectal OR Rectal Neoplasm OR Rectum Neoplasms OR Neoplasm, Rectum OR Rectum Neoplasm OR Rectal Tumors OR Rectal Tumor OR Tumor, Rectal OR Neoplasms, Rectal OR Cancer of Rectum OR Rectum Cancers OR Rectal Cancer OR Cancer, Rectal OR Rectal Cancers OR Rectum Cancer OR Cancer, Rectum OR Cancer of the Rectum):ab,ti,kw | 8772 |
| #3 | (Colorectal Neoplasms OR Colorectal Neoplasm OR Neoplasm, Colorectal OR Neoplasms, Colorectal OR Colorectal Tumors OR Colorectal Tumor OR Tumor, Colorectal OR Tumors, Colorectal OR Colorectal Cancer OR Cancer, Colorectal OR Cancers, Colorectal OR Colorectal Cancers OR Colorectal Carcinoma OR Carcinoma, Colorectal OR Carcinomas, Colorectal OR Colorectal Carcinomas):ab,ti,kw | 19796 |
| #4 | (Colonic Neoplasm OR Neoplasm, Colonic OR Neoplasms, Colonic OR Colon Neoplasms OR Colon Neoplasm OR Neoplasm, Colon OR Neoplasms, Colon OR Cancer of Colon OR Colon Cancers OR Colon Cancer OR Cancer, Colon OR Cancers, Colon OR Cancer of the Colon OR Colonic Cancer OR Cancer, Colonic OR Cancers, Colonic OR Colonic Cancers OR Colon Adenocarcinoma OR Adenocarcinoma, Colon OR Adenocarcinomas, Colon OR Colon Adenocarcinomas):ab,ti,kw | 8931 |
| #5 | (Chemotherapy OR Therapy, Drug OR Drug Therapies OR Therapies, Drug OR Chemotherapy OR Chemotherapies OR Pharmacotherapy OR Pharmacotherapies):ti,ab,kw | 561536 |
| #6 | (Prospective Studies OR Prospective Study OR Studies, Prospective OR Study, Prospective OR Prospective Comparative Study OR Prospective):ab,ti,kw | 257223 |
| #7 | (Retrospective Studies OR Studies, Retrospective OR Study, Retrospective OR Retrospective Study):ab,ti,kw | 37244 |
| #8 | (randomized controlled trial OR controlled clinical trial OR randomized OR placebo OR clinical trials as topic OR randomly OR trial):ab,ti,kw | 1457611 |
| #9 | #2 OR #3 OR #4 | 29182 |
| #10 | #1 AND #9 | 365 |
| #11 | #6 OR #7 OR #8 | 1489496 |
| #12 | #10 AND #11 | 317 |
